# Functional Analysis of *LTS-PYL* in Modulating Plant Drought Responses

**DOI:** 10.3390/antiox15020178

**Published:** 2026-01-30

**Authors:** Rahmatullah Jan, Sajjad Asaf, Saleem Asif, Zakirullah Khan, Lubna, Eman R. Elsharkawy, Syed Abdullah Gilani, Kyung-Min Kim

**Affiliations:** 1 Coastal Agriculture Research Institute, Kyungpook National University, Daegu 41566, Republic of Korea; rahmat2021@knu.ac.kr; 2 Natural and Medical Science Research Center, University of Nizwa, Nizwa 616, Oman; 3 Department of Applied Biosciences, Graduate School, Kyungpook National University, Daegu 41566, Republic of Korea; 4 Center for Health Research, Northern Border University, Arar 73213, Saudi Arabia; 5 Department of Biological Sciences and Chemistry (DBSC), College of Arts and Sciences (CAS), University of Nizwa, Nizwa 616, Oman

**Keywords:** ABA signaling, antioxidant defense, CRISPR-Cas9, drought tolerance, *LTS-PYL*

## Abstract

Drought severely limits plant productivity, and understanding its regulatory mechanisms remains essential. Here, we characterize Lipid Transport Superfamily-Polyketide cyclase/dehydrase (*LTS-PYL*), a PYR/PYL/RCAR-domain gene, using Arabidopsis overexpression and CRISPR-Cas9 genome-edited lines to elucidate its role in drought adaptation. *LTS-PYL* overexpression enhanced early seedling growth, increasing root length (RL) by 40% and 31%, whereas genome-edited lines exhibited severe defects, including 42%, 28% reductions in fresh weight and 63%, 50% decreases in root length relative to WT-T. Under drought stress, overexpression lines displayed strong growth and reproductive resilience, with shoot length (SL) increased by up to 80%, silique length (Sil L) by 61%, and seed number doubled compared with WT-T. In contrast, genome-edited lines showed marked reductions in these traits, confirming their drought sensitivity. *LTS-PYL* overexpression strongly suppressed oxidative stress, reducing H_2_O_2_ by 74% and 68% and O_2_^−^· by 39% and 38%, while increasing relative water content (RWC) by 42% and 39%. Genome-edited lines exhibited elevated (H_2_O_2_, O_2_^−^·) and up to 33% lower RWC. Antioxidant capacity was also strengthened in overexpression plants, with catalase (CAT) and peroxidase (POD) activities increasing by 138%, 168% and 62%, 148%, and malondialdehyde (MDA) and electrolyte leakage (EL) reduced by 23%, 37%, relative to WT-T. Conversely, genome-edited lines showed weakened antioxidant defenses and higher membrane damage. Transcriptionally, overexpression activated drought-responsive genes, elevating *LTS-PYL* (604%, 472%), *DREB2A* (227%, 200%), and ABA levels (48%, 34%), whereas genome-edited lines showed strongly reduced expression and ABA decreases of 66%, 62%. Additionally, *LTS-PYL* enhanced osmotic adjustment, increasing proline (58%, 53%), sugars (37%, 46%), and sucrose (111%, 100%), while limiting chlorophyll (Chl) loss to 9%, 20%. Genome-edited lines exhibited reduced osmolytes and severe chlorophyll decline. Overall, *LTS-PYL* acts as a strong positive regulator of drought tolerance, integrating ABA signaling, osmotic adjustment, ROS detoxification, and transcriptional activation.

## 1. Introduction

Drought stress is one of the most severe environmental constraints inhibiting plant growth, development, and productivity worldwide. Reduced water availability affects multiple physiological and biochemical processes, finally resulting in impairing photosynthesis, disrupting metabolic homeostasis, and reducing crop yield. In response to drought, plants activate a complex array of adoptive mechanisms that include stomatal regulation, osmolytes accumulation, modulation of antioxidant capacity, and induction of stress responsive genes. The efficiency of these strategies largely determines the plant’s ability to maintain cellular integrity and survive prolonged period of water deficit.

Drought-induced reactive oxygen species (ROS), including superoxide anion (O_2_^•–^), hydrogen peroxide (H_2_O_2_), and the lipid peroxidation marker malondialdehyde (MDA), can damage cellular membranes and DNA and promote oxidative degradation of essential biomolecules [1]. Plants mitigate this oxidative burden by activating both enzymatic and non-enzymatic antioxidant system, such as superoxide dismutase (SOD), peroxidase (POD), and catalase (CAT), which work collectively to restore redox homeostasis [1,2]. In parallel, transcription factors play a critical role in linking drought perception to downstream molecular responses. Among these, *DREB* proteins, members of the ERF family, are key regulators functioning primarily through ABA-independent pathways. Enhanced expression of *DREB* genes has been shown to significantly strengthen drought tolerance and improve yield under water-limited conditions [3]. For example, *GmDREB2A* from soybean is strongly induced by dehydration, heat, and low-temperature stress, underscoring its importance in broad-spectrum stress responses [4]. Additionally, ABA biosynthetic genes such as *AO3* and *ABA3* contribute to drought adaptation by modulating ABA accumulation and controlling stomatal behavior during water deficit [5].

Abscisic acid (ABA) signaling cascade represents one of the central hormonal pathways regulating plant adaptation to environmental stress. ABA regulates a variety of physiological processes such as stomatal closure, root system modulation, enhances osmotic adjustment, and coordinates transcriptional reprogramming during drought stress [2]. It is well established that ABA perception begins with its binding to the PYRABACTIN RESISTANCE1 (PYR1)/PYR1-Like (PYL)/REGULATORY COMPONENTS OF ABA RECEPTORS (RCAR) (PYR/PYL/RCAR) receptor family, which initiates the core ABA signaling cascade by modulating the activity of clade A protein phosphatases 2C (PP2Cs) and subsequently activating sucrose non-fermenting related protein kinases 2 (*SnRK2s*) [6,7,8]. Upon ABA precipitation, *PYL* receptors inhibit *PP2Cs*, allowing *SnRK2s* to activate downstream transcriptional factors and stress responsive genes [9]. Studies have demonstrated that *PYL* family members positively regulate drought-stress tolerance by enhancing ABA sensitivity, ROS scavenging capacity, and protective metabolite accumulation [10,11].

Despite the growing evidence supporting the involvement of PYL receptors in abiotic stress adaptation, the specific role of the *LTS-PYL* gene remains largely uncharacterized. Preliminary observations suggest that this receptor may act as a positive regulator of drought and ABA responses, yet its detailed molecular, physiological, and biochemical have not been elucidated. Importantly, both overexpression and genome editing via CRISPR-Cas9 approaches are powerful tools for dissecting the contribution of individual *PYL* genes to drought-stress tolerance.

To overcome these knowledge gaps, we generated *Arabidopsis thaliana* plants overexpressing *LTS-PYL* as well as CRISPR/Cas9-mediated genome edited lines. These materials allowed us to investigate how altered *LTS-PYL* expression influences plants’ performance under drought conditions. We specifically evaluated agronomic and morphological traits, oxidative stress markers, antioxidant defense responses, osmolyte accumulation, and the expression of key drought and ABA responsive genes. Therefore, the main objective of this study was to elucidate the functional role of *LTS-PYL* in drought-stress tolerance in *Arabidopsis thaliana*. By combining gene overexpression and CRISPR Cas9-mediated editing, we aimed to determine how changes in *LTS-PYL* expression influence plant growth, oxidative and antioxidant dynamics, osmolyte accumulation, and transcriptional responses to drought and ABA. This work provides new insights into the regulatory functions of *PYL* receptors and highlights the potential of *LTS-PYL* as a promising target for improving drought-stress tolerance in plants.

## 2. Materials and Methods

### 2.1. Generation of Transgenic Arabidopsis Plants Overexpressing LTS-PYL

*Arabidopsis thaliana* (Col-0) overexpression lines for *LTS-PYL* were generated by first amplifying the full-length coding region of *LTS-PYL* (Locus: AT4G32870, details are provided in Supplemental File S1) and inserted into the pCR8/GW/TOPO entry vector (provided in Supplemental File S3), which carries spectinomycin resistance. The construct was introduced into *Escherichia coli* DH5α via heat-shock transformation, and positive colonies were verified through colony PCR followed by sequencing. The confirmed entry clone was subsequently transferred into the Gateway-compatible binary vector pEarleyGate103, harboring a CaMV 35S promoter and kanamycin resistance (provided in Supplemental File S4), via an LR recombination reaction to achieve constitutive transgene expression. All resulting plasmids were validated by commercial sequencing prior to plant transformation. The finalized overexpression construct was electroporated into *Agrobacterium tumefaciens* strain GV3101, which was then used to transform Arabidopsis plants using the floral dip method [12]. Transgenic seedlings were selected by spraying 1-week-old progeny with 5 µg/mL Basta, and herbicide-resistant T1 lines were identified for further characterization.

### 2.2. Generation of LTS-PYL-Edited Arabidopsis via CRISPR/Cas9

To obtain *Arabidopsis thaliana* lines carrying targeted mutations in *LTS-PYL*, two sgRNAs were designed using the CRISPR RGEN design platform. The sequences of sgRNAs are provided in Supplemental File S2. Each sgRNA was assembled into the CRISPR/Cas9 expression vector pRGEB32 (provided in Supplemental File S5), which contains the Cas9 nuclease, by inserting the guide sequences downstream of the U3 promoter through BsaI-based Golden Gate cloning [13]. Sequence analysis was performed to verify the correct incorporation of each guide RNA. The validated CRISPR constructs were introduced into *Agrobacterium tumefaciens* strain EHA105 using heat-shock transformation and subsequently delivered into *Arabidopsis* plants through the standard floral dip procedure. Putative T1 transformants were confirmed through genotypic analysis to identify individuals carrying CRISPR-induced edits at the *LTS-PYL* locus. Genome-edited lines confirmed through molecular analysis were selected for downstream characterization.

### 2.3. Molecular Confirmation and Sequencing of CRISPR-Edited Arabidopsis Lines

Genomic DNA was isolated from putative genome-edited Arabidopsis plants using the DNeasy Plant Mini Kit (QIAGEN, Cat. 69104, Hilden, Germany) according to the manufacturer’s protocol. The presence of T-DNA was verified by PCR amplification of the hygromycin resistance marker using the Hyro-F and Hygro-R primers. PCR reactions were carried out with high-fidelity Pfu polymerase in combination with the Genetbio PCR Master Mix (Yuseong-gu, Daejeon, Republic of Korea), following the recommended guidelines. To assess mutations introduced by CRISPR/Cas9, the coding region of *LTS-PYL* was amplified using gene-specific primers, and the resulting products were subjected to Sanger sequencing. Sequences were compared against the NCBI reference sequence to identify insertions, deletions, or other sequence alterations at the target site.

### 2.4. Experimental Design

In this study, four treatment groups were used: wild-type control (CK), wild-type treated (WT-T), *LTS-PYL* overexpressor treated (*OELTS-PYL-T*), and genome-edited treated (*gelts-pyl-t*). For both the overexpression and genome-edited lines, two independent genotypes were included (*OELTS-PYL-T1* and *T2*; *gelts-pyl-t1* and *t2*). All *Arabidopsis thaliana* plants belonged to the ecotype Col-0. Drought stress was induced in thirty-day-old plants using 20% PEG 6000 (Burlington, MA, USA), which was applied to the soil under controlled growth conditions (16/8 h light/dark cycle, 25 °C, and 60% relative humidity), following the method described by [14]. Prior to stress application, all treatment groups were irrigated with normal water for thirty days. After this period, drought-stress treatments received irrigation with 20% PEG 6000, while the control group continued to receive normal water. Plants were monitored regularly, and leaf samples were collected one week after drought induction. The collected samples were immediately frozen in liquid nitrogen and stored at −80 °C for further analyses.

### 2.5. Assessment of H_2_O_2_, O_2_^−^·, and Leaf Relative Water Content

The accumulation of superoxide anion (O_2_^−^·), hydrogen peroxide (H_2_O_2_), and relative water content (RWC) was measured after one week of drought treatment. All experiments were performed in two biological replicates. The quantification of O_2_^−^· and H_2_O_2_ was carried out following the methods described in our previous study [15]. For RWC determination, fully expanded leaves were collected in two replicates after one week of drought stress. Leaf disks (5 mm in diameter) were excised, and their fresh weight (FW) was recorded immediately. The disks were then submerged in 10 mL of distilled water for 3 h to obtain the turgid weight (TW). Following this, samples were dried at 70 °C for 24 h to measure the dry weight (DW). Relative water content was calculated using the formula:

(1) $$RWC\;\left(\%\right)=\frac{Fw-DW}{TW-DW}\times 100$$

### 2.6. Measurement of CAT, POD, SOD, and MDA

Catalase (CAT), peroxidase (POD), and superoxide dismutase (SOD) activities were measured using fresh leaves collected after one week of drought exposure, following the detailed procedures described in our previous study [16]. Malondialdehyde (MDA) content, an indicator of lipid peroxidation, was quantified using an MDA Assay Kit (Sigma, Burlington, MA, USA) according to the manufacturer’s instructions [17]. Briefly, 10 mg of leaf tissue was homogenized on ice in 300 µL of MDA lysis buffer supplemented with 3 µL of BHT. The homogenate was centrifuged at 13,000 rpm for 10 min, and the supernatant was collected. A 200 µL aliquot of the supernatant was transferred to a 1 mL microcentrifuge tube, mixed with 600 µL of TBA reagent, and incubated at 95 °C for 1 h. Samples were then cooled on ice for 10 min. Finally, 200 µL of each sample and blank were loaded into a 96-well microplate, and absorbance was measured at 532 nm using a Shimadzu spectrophotometer (Shimadzu, Kyoto, Japan). Each reaction was performed in two replicates. MDA concentration was calculated using the following formula:

(2) $$C=\frac{S_{a}}{S_{v}}\times \;D$$

where *C* is the MDA content, *S_a_* is the amount of MDA in the sample, *S_v_* is the sample volume, and *D* is the dilution factor.

### 2.7. ABTS and DPPH Radical Scavenging Activity Analysis

Antioxidant capacity was assessed using ABTS and DPPH scavenging assays one week after drought exposure. The ABTS assay was performed following our previous procedure with minor modifications [15]. Stock solution of 7 mM ABTS and 2.4 mM potassium persulfate were prepared in double distilled water and mixed in equal volumes to generate ABTS radical. The moisture was incubated in the dark for 12 h. The resulting ABTS-radical-containing solution was diluted (1 mL in 20 mL water) to obtain the absorbance of approximately 0.7 at 734 nm. Leaf extracts were mixed with 180 µL of the diluted ABTS radical solution, and absorbance was recorded at 734 nm using spectrophotometer (Thermo Fisher Scientific, Vantaa, Finland). Radical scavenging activity was calculated using following formula:

(3) $$\mathrm{ABTS}\;\mathrm{Scavenging}\;\mathrm{Activity}\;\left(\%\right)\;=\left(\frac{A_{c}-A_{s}}{A_{c}}\right)\;\times 100$$

where *A*_(_*_C_*_)_ is the absorbance of the control and *A*_(_*_S_*_)_ is the absorbance of the sample mixture.

DPPH assay was conducted using 0.1% DPPH solution, prepared in methanol. A 100 µL of plant extract and DPPH solution were mixed in 96-well microplate and incubated for 30 min at 25 °C. The control reaction contained 100 µL of DPPH solution and 100 µL of methanol. Absorbance was measured at 517 nm using the same spectrophotometer and DPPH scavenging activity was calculated by using following formula:

(4) $$DPPH\;Scavenging\;Activity\;\left(\%\right)\;=[1-\frac{\left(A-A_{0}\right)}{\left(B-B_{0}\right)}]\times 100$$

A represents the absorbance of the DPPH + sample mixture, *A*_0_ the absorbance of methanol with the sample, *B* the absorbance of DPPH with methanol (control), and *B*_0_ the absorbance of methanol alone.

### 2.8. Electrolyte Leakage Assessment

Electrolyte leakage was assessed after one week of drought exposure using the method described in our previous study [16]. Fresh leaves were collected, and 100 mg of leaf tissue was cut into uniformly sized pieces and placed in test tubes containing 10 mL of deionized distilled water. The tubes were sealed and incubated at 32 °C for 2 h to allow electrolyte release into the solution. The initial electrolyte conductivity (EC_1_) was then recorded using a conductivity meter (Model CM-115, Kyoto, Japan). Subsequently, the samples were autoclaved at 120 °C for 20 min to completely lyse the tissues, cooled to room temperature, and the final conductivity (EC_2_) was measured. Electrolyte leakage was calculated using the following formula:

(5) $$Electrolte\;Leakage\;\left(\%\right)=\frac{EC1}{EC2}\times 100$$

### 2.9. RNA Extraction, cDNA Preparation, and qRT-PCR Analysis

Total RNA was purified from Arabidopsis samples using the RNeasy Plant Mini Kit (Qiagen, Valencia, CA, USA) following the manufacturer’s guidelines. Two micrograms of high-quality RNA were subsequently used as template for first-strand cDNA synthesis with the qPCR-Bio cDNA Synthesis Kit (PCRBIOSYSTEM, Seoul, Republic of Korea). Quantitative real-time PCR was conducted on a StepOne™ Plus system (Thermo Fisher Scientific, Seoul, Republic of Korea) using a SYBR Green–based 2× Real-Time PCR Master Mix (BIOFACT, Daejeon, Republic of Korea). Expression levels of target genes were normalized to *ACTIN*, which served as the internal reference, and relative transcript abundance was calculated using the 2^−ΔΔCt^ method. The primer sequences for the selected genes are provided in Supplemental File S2.

### 2.10. Analysis of ABA, Proline, Soluble Sugars, Sucrose, and Chlorophyll Levels

Fresh leaf samples were collected after one week of drought treatment to quantify abscisic acid (ABA) levels. ABA extraction and measurement were performed using the Plant Abscisic Acid ELISA Kit (LifeSpan BioSciences, Seattle, WA, USA) following the manufacturer’s instructions.

Proline content was also assessed in leaf tissues after one week of drought exposure using the method described in [18]. Briefly, 300 mg of fresh leaves were ground in liquid nitrogen and extracted in 3% sulfosalicylic acid. The homogenate was heated in a boiling water bath for 10 min and subsequently cooled at room temperature for 15 min. After centrifugation at 5000 rpm for 10 min, the resulting supernatant was mixed with 2.5% ninhydrin and glacial acetic acid and incubated at 100 °C for 1 h. The reaction was immediately stopped on ice, followed by the addition of 4 mL toluene. Absorbance was recorded at 520 nm using a spectrophotometer, and proline concentration was calculated as µg g^−1^ fresh weight.

Total sugar and sucrose contents were determined from freeze-dried leaves harvested after one week of drought stress, following the procedure outlined in [19]. Lyophilized tissue (0.5 g) was ground in liquid nitrogen, extracted with 2 mL of 80% ethanol, and incubated at 80 °C for 20 min. The mixture was centrifuged at 10,000 rpm for 15 min, and the residue was discarded. The pellet was then resuspended in 4 mL of distilled water and filtered through a 0.2 µm membrane. Sugar and sucrose levels were quantified using HPLC equipped with a Bio-Rad Aminex 87C column (300 × 7.8 mm) (Hercules**,** CA, USA), with distilled water as the mobile phase at a flow rate of 0.6 mL min^−1^.

Chlorophyll content was assessed using a SPAD meter (SPAD-502 Plus; Konica Minolta Sensing, Seoul, Republic of Korea). Measurements were taken from the tip and middle regions of five leaves per treatment.

### 2.11. Statistical Analysis

The experiment was conducted using a completely randomized design with two biological replicates. Data were analyzed using one-way analysis of variance (ANOVA), followed by Tukey’s post hoc test. Results are presented as mean ± standard deviation. All graphs were generated using GraphPad Prism 10 (GraphPad Software, San Diego, CA, USA).

## 3. Results

### 3.1. Functional Analysis of LTS-PYL in Arabidopsis

**Figure 1:**
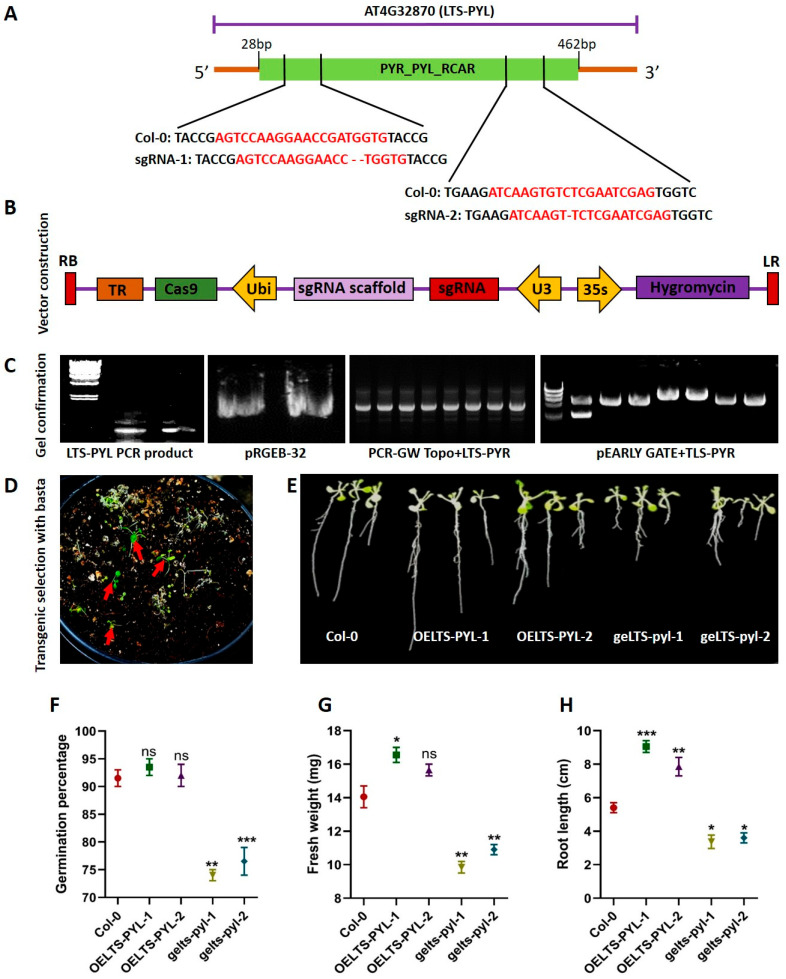
Functional characterization of *LTS-PYL* in Arabidopsis. (**A**) Schematic representation of the *LTS-PYL* gene structure showing the PYR_PYL_RCAR domain target sites of sgRNAs. (**B**) Structure of the CRISPR-Cas9vector containing UBI-Cas9 cassette, sgRNA scaffold, U3 promoter, and hygromycin resistant marker. (**C**) PRC confirmation of *LTS-PYR* amplification, pRGEB-32 colonies, GW-TOPO recombinant clones, and final pEARLY GATE-*LTS-PYL* constructs. (**D**) Selection of T1 transgenic seedlings after Basta foliar application (red arrows indicate resistant plants). (**E**) Representative 7-day-old seedlings of Col-0, overexpression lines (*OELTS-PYL-1/2*), and genome edited lines (*gelts-pyl-1/2*). (**F**–**H**) Quantification of germination percentage (**F**), fresh weight (**G**), and root length (**H**). DATA represent mean ± SD (n = 3). Asterisks indicate significant differences from Col-0 and WT-T (* *p* < 0.05, ** *p* < 0.01, *** *p* < 0.001); ns = not significant.

To functionally characterize *LTS-PYL*, two sgRNAs were designed to specifically target the PYR_PYL_RCAR domain of the gene (Figure 1A). The CRISPR–Cas9 construct, carrying Cas9 under the Ubi promoter, the sgRNA cassette, and a hygromycin resistance marker, was assembled as shown in Figure 1B. Successful amplification of the full-length *LTS-PYL* sequence, insertion of the sgRNA into pRGEB-32, generation of GW-TOPO recombinants, and construction of the final pEARLY-GATE vector were all confirmed by PCR and gel electrophoresis (Figure 1C).

Transformed T1 seedlings of the overexpression lines were screened using the Basta application, and resistant individuals were selected (Figure 1D). Phenotypic evaluation of 7-day-old seedlings revealed clear differences among the genotypes (Figure 1E). Germination percentage did not differ between Col-0 and *OELTS-PYL* lines; however, both knockout lines (*gelts-pyl-1* and *gelts-pyl-2*) showed significantly decreased germination compared to Col-0 (Figure 1F). Seedling fresh weight increased by 17% in *OELTS-PYL-1*, remained similar to Col-0 in *OELTS-PYL-2*, and decreased sharply in *gelts-pyl-1* (42% reduction) and *gelts-pyl-2* (28% reduction) (Figure 1G). Similarly, root length increased by 40% in *OELTS-PYL-1* and 31% in *OELTS-PYL-2*, while it was reduced by 63% in *gelts-pyl-1* and 50% in *gelts-pyl-2* compared with Col-0 (Figure 1H).

### 3.2. LTS-PYL Enhances Vegetative and Reproductive Performance Under Drought Stress

**Figure 2:**
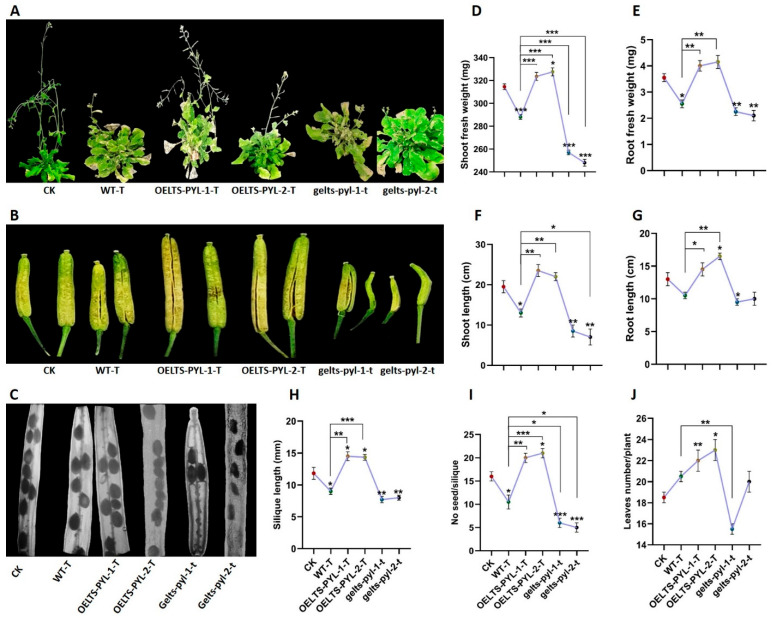
Effect of *LTS-PYL* overexpression and genome editing on vegetative and reproductive traits in Arabidopsis. (**A**) Represent morphology of mature plants. (**B**) Siliques from each genotype. (**C**) Seed distribution and silique structure. (**D**–**J**) Quantitative analysis of shoot fresh weight (**D**), root fresh weight (**E**), shoot length (**F**), root length (**G**), silique length (**H**), seed number per silique (**I**), and leaf number per plant (**J**). DATA represent mean ± SD (n = 3). Asterisks indicate significant differences from Col-0 and WT-T (* *p* < 0.05, ** *p* < 0.01, *** *p* < 0.001).

To investigate the role of *LTS-PYL* in plant performance under drought stress, we evaluated the vegetative and reproductive traits of wild-type (WT), overexpression (*OELTS-PYL-1-T* and *OELTS-PYL-2-T*), and genome-edited knockout lines (*gelts-pyl-1* and *gelts-pyl-2*) under drought treatment (Figure 2). As expected, drought-stressed WT plants (WT-T) exhibited reduced growth compared with well-watered control plants (CK). In contrast, both OELTS lines maintained stronger growth and greener foliage under drought stress, whereas the gelts lines showed severe growth retardation and premature leaf senescence (Figure 2A).

Drought stress markedly affected silique development in WT plants, resulting in shorter siliques compared with CK. However, *OELTS-PYL-1-T* and *OELTS-PYL-2-T* produced longer siliques with normal seed filling even under drought conditions. In contrast, genome edited lines developed extremely short, shriveled siliques with poor seed set (Figure 2B, C).

Quantitative measurements supported these observations. Shoot fresh weight decreased in WT-T compared with CK, whereas both OELTS lines retained significantly higher shoot biomass under drought (Figure 2D). Conversely, the *gelts-pyl-1* and *gelts-pyl-2* mutants showed drastic reductions in shoot fresh weight, with *gelts-pyl-2* being the most drought sensitive. Root fresh weight followed a similar trend: *OELTS-PYL-1-T* and *OELTS-PYL-2-T* maintained comparatively high root biomass, while both genome-edited lines exhibited sharp declines under drought (Figure 2E).

Shoot and root lengths were also strongly influenced by drought stress. WT-T plants showed clear reductions in both traits relative to CK. However, *OELTS-PYL-1-T* and *OELTS-PYL-2-T* maintained significantly longer shoots (80% and 69%) and roots (38% and 57%) under drought, compared with WT-T (Figure 2F, G). In contrast, *gelts-pyl-1* and *gelts-pyl-2* displayed severe decreases in shoot and root length.

Reproductive traits followed the same pattern of *LTS-PYL*-dependent drought tolerance. Silique length decreased 23% in WT-T, but increased significantly in *OELTS-PYL-1-T* (61%) and *OELTS-PYL-2-T* (59%) relative to WT-T. In contrast, *gelts-pyl-1* and *gelts-pyl-2* showed silique length reductions of 14% and 11%, respectively (Figure 2H). Seed number per silique was likewise enhanced in *OELTS-PYL-1-T* and *OELTS-PYL-2-T* by 90% and 100% relative to WT-T, whereas both knockout lines produced drastically fewer seeds, with *gelts-pyl-2* being the most severely affected (Figure 2I).

Leaf number also varied among genotypes under drought. WT-T plants exhibited a 20% reduction relative to CK, while in contrast, *OELTS-PYL-1-T* and *OELTS-PYL-2-T* displayed 10% and 18% increases in leaf number compared with CK plants (Figure 2J).

### 3.3. LTS-PYL Modulates ROS Accumulation and Water Retention Under Drought Stress

**Figure 3:**
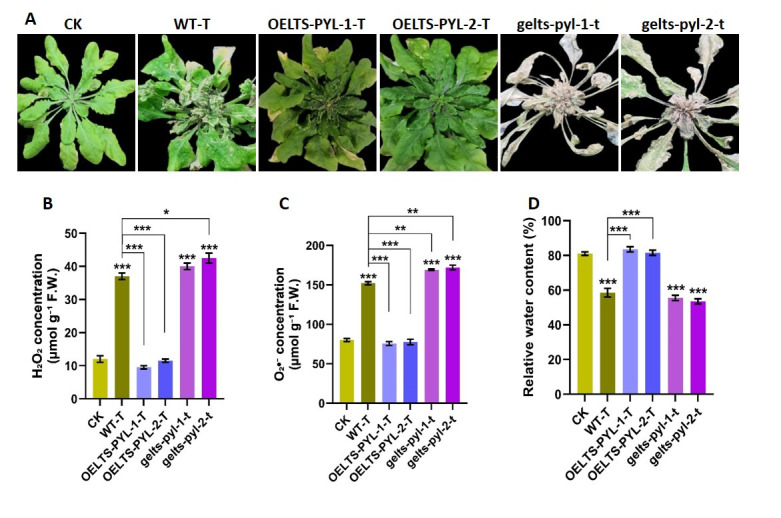
*LTS-PYL* regulates ROS accumulation and water status under drought stress. (**A**) Representative image of WT control (WT-C), drought-stressed WT (WT-T), *LTS-PYL* over expression lines (*OELTS-PYL-1-T* and *OELTS-PYL-2-T*), and genome-edited line (*gelts-pyl-1* and *gelts-pyl-2*) after drought stress. (**B**) Hydrogen peroxide (H_2_O_2_) contents. (**C**) Superoxide anion (O_2_^−^·) contents. (**D**) Relative water contents. DATA represent mean ± SD (n = 3). Asterisks indicate significant differences from Col-0 and WT-T (* *p* < 0.05, ** *p* < 0.01, *** *p* < 0.001).

To further assess the physiological role of *LTS-PYL* under drought stress, we quantified reactive oxygen species (ROS), including H_2_O_2_ and O_2_^−^·, as well as relative water contents in WT, *LTS-PYL* overexpression lines, and genome-edited lines (Figure 3). Visual observations showed that drought stress caused severe leaf bleaching and tissue damage in *gelts-pyl-1* and *gelts-pyl-2*, followed by WT-T, whereas *OELTS-PYL-1-T* and *OELTS-PYL-2-T* maintained greener and healthier foliage (Figure 3A). Drought stress significantly increased H_2_O_2_ and O_2_^−^· accumulation in WT-T, *gelts-pyl-1*, and *gelts-pyl-2* compared with CK (Figure 3B, C). In contrast, *OELTS-PYL-1-T* and *OELTS-PYL-2-T* exhibited no significant increase in ROS level under drought stress. Instead, both lines (*OELTS-PYL-1-T* and *OELTS-PYL-2-T*) markedly reduced ROS accumulation, with H_2_O_2_ level decreased by 74 and 68% and O_2_^−^· by 39 and 38%, respectively, compared with WT-T. Relative water contents were significantly reduced under drought, declining by 27% in WT-T, 31% in *gelts-pyl-1*, and 33% *gelts-pyl-2* compared with CK (Figure 3D). However, *OELTS-PYL-1-T* and *OELTS-PYL-2-T* displayed significantly improved water retention, showing 42 and 39% higher RWC, respectively, compared with WT-T. These results demonstrate that *LTS_PYL* enhances drought tolerance in Arabidopsis by suppressing ROS accumulation and maintaining higher water content, whereas loss of *LTS-PYL* intensifies oxidative stress and reduces water status under drought stress.

### 3.4. LTS-PYL Modulates Antioxidant Defense and Membrane Stability Under Drought Stress

**Figure 4:**
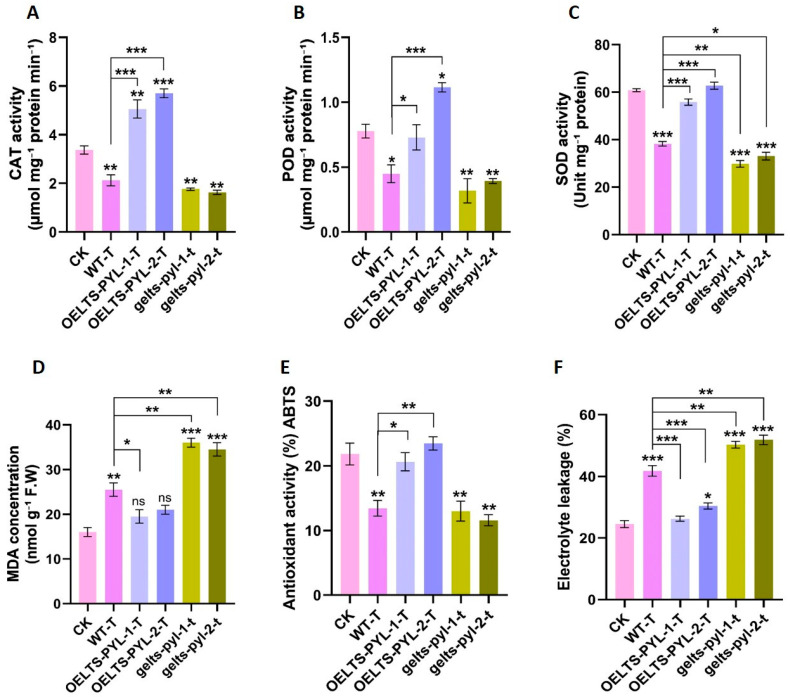
*LTS-PYL* modulates antioxidant enzymes activities and membrane stability under drought stress. (**A**–**C**) Activities of CAT, POD, SOD enzymes, respectively. (**D**) MDA accumulation as a marker of lipid peroxidation. (**E**) ABTS scavenging activity. (**F**) Electrolyte leakage percentage indicating membrane damage. DATA represent mean ± SD (n = 3). Asterisks indicate significant differences from Col-0 and WT-T (* *p* < 0.05, ** *p* < 0.01, *** *p* < 0.001). ns = not significant.

To evaluate the antioxidant responses of the different *LTS-PYL* genotypes under drought stress, we quantified the activities of major ROS-scavenging enzymes along with indicators of membrane damage (Figure 4). CAT activity increased significantly in both overexpression lines but decreased in WT-T and the genome-edited lines compared with CK. Relative to WT-T, CAT activity was enhanced by 138% and 168% in *OELTS-PYL-1-T* and *OELTS-PYL-2-T*, respectively, whereas it declined by 16% and 23% in *gelts-pyl-1-t* and *gelts-pyl-2-t* (Figure 4A). Similarly, POD activity was strongly induced in the *OELTS-PYL-2-T* line but was significantly reduced in WT-T and both genome-edited lines compared with CK. Compared with WT-T, POD activity increased by 62% and 148% in *OELTS-PYL-1-T* and *OELTS-PYL-2-T*, while it decreased by 29% and 12% in *gelts-pyl-1-t* and *gelts-pyl-2-t*, respectively (Figure 4B). SOD activity followed a similar pattern, although overexpression lines showed no significant change relative to CK, WT-T, and both genome-edited lines displayed significant reductions (Figure 4C). However, both overexpression lines showed higher SOD activity, while the genome-edited lines showed reduced activity compared with WT-T.

In contrast, MDA levels (an indicator of lipid peroxidation) were significantly elevated in WT-T and genome-edited lines, whereas only slight, non-significant increases were observed in the overexpression lines relative to CK (Figure 4D). Specifically, *OELTS-PYL-1-T* reduced MDA content by 23%, while *gelts-pyl-1-t* and *gelts-pyl-2-t* showed 41% and 35% increases relative to WT-T plants. Non-enzymatic antioxidant capacity, measured by ABTS activity, decreased sharply in WT-T and genome-edited lines compared with CK (Figure 4E). However, *OELTS-PYL-1-T* and *OELTS-PYL-2-T* exhibited significant increases of 53% and 74%, respectively, relative to WT-T, whereas the genome-edited lines showed responses similar to WT-T.

Consistently, drought-stress impaired membrane stability across all genotypes, as indicated by higher electrolyte leakage relative to CK (Figure 4F). However, membrane integrity was better maintained in the overexpression lines, where electrolyte leakage was reduced by 37% in *OELTS-PYL-1-T* and 27% in *OELTS-PYL-2-T*. In contrast, leakage increased by 20% and 24% in *gelts-pyl-1-t* and *gelts-pyl-2-t* relative to WT-T. Collectively, these findings demonstrate that *LTS-PYL* overexpression markedly enhances antioxidant defense and membrane protection under drought stress, whereas loss of *LTS-PYL* function compromises drought tolerance.

### 3.5. LTS-PYL Regulation of Gene Expression and ABA Responses Under Drought

**Figure 5:**
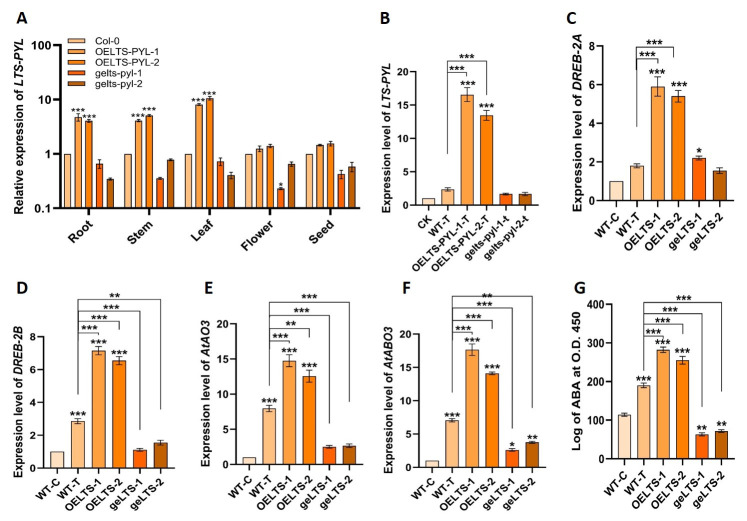
Expression profiling of *LTS-PYL* and key ABA and stress-responsive genes in Arabidopsis. (**A**) Tissue-specific expression of *LTS-PYL* in Col-0, overexpression, and genome edited lines under normal (control) condition. (**B**–**F**) qRT-PCR analysis of *LTS*, *DREB2A*, *DREB2B*, *AtAO3*, *AtABO3* expression in all genotypes. (**G**) ABA accumulation. DATA represent mean ± SD (n = 3). Asterisks indicate significant differences from Col-0 and WT-T (* *p* < 0.05, ** *p* < 0.01, *** *p* < 0.001).

To investigate the regulatory role of *LTS-PYL* in Arabidopsis, we examined its tissue-specific expression and assessed drought-induced transcriptional responses in overexpression and genome-edited lines (Figure 5). *LTS-PYL* displayed clear organ-specific expression, with both overexpression lines showing markedly higher transcript levels in the root, stem, and leaf tissues compared with Col-0, with the strongest induction observed in leaves (Figure 5A). In contrast, the genome-edited lines consistently exhibited reduced expression across all tissues.

Under drought stress, *LTS-PYL* expression was strongly enhanced in the overexpression lines, with *OELTS-PYL-1-T* and *OELTS-PYL-2-T* showing 604% and 472% increases, respectively, compared with WT-T, whereas both genome-edited lines remained substantially lower than WT-T (Figure 5B). Key drought-responsive genes also showed robust induction in the overexpression backgrounds. *DREB2A* transcripts increased by 227% and 200%, and *DREB2B* by 150% and 129%, in *OELTS-PYL-1-T* and *OELTS-PYL-2-T*, respectively (Figure 5C,D). Except for a slight induction of *DREB2A* in *gelts-pyl-1-T*, both edited lines exhibited overall reductions in *DREB2A* and *DREB2B* expression relative to WT-T.

Similarly, drought stress elevated the expression of *AtAO3* and *AtABO3* in all genotypes compared with CK (Figure 5E,F). However, the genome-edited lines displayed significantly lower transcript levels than WT-T. In contrast, overexpression enhanced these ABA-related genes, with *AtAO3* increasing by 85% and 57%, and *AtABO3* by 150% and 100%, in *OELTS-PYL-1-T* and *OELTS-PYL-2-T*, respectively, relative to WT-T. Consistent with transcriptional activation, ABA accumulation increased in WT-T and both overexpression lines, whereas it declined sharply in genome-edited plants compared with CK. Relative to WT-T, ABA levels increased 48% and 34% in *OELTS-PYL-1-T* and *OELTS-PYL-2-T*, but decreased 66% and 62% in *gelts-pyl-1-T* and *gelts-pyl-2-T*, respectively (Figure 5G). These results demonstrate that *LTS-PYL* positively regulates drought-responsive transcription factors and ABA biosynthesis genes, enhancing ABA accumulation and stress responsiveness, while loss of *LTS-PYL* function compromises these adaptive responses.

### 3.6. Osmolyte Accumulation and Chlorophyll Retention in LTS-PYL Lines Under Drought

**Figure 6:**
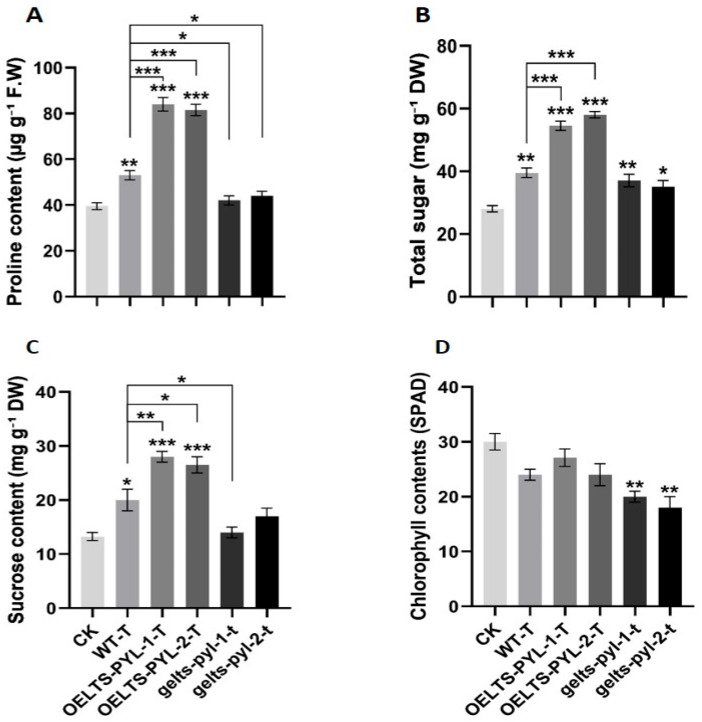
*LTS-PYL* regulates osmolyte accumulation and chlorophyll content in Arabidopsis under drought stress. (**A**) Proline content, (**B**) Sugar content, (**C**) Succrose content, (**D**) Chlorophyll content. DATA represent mean ± SD (n = 3). Asterisks indicate significant differences from Col-0 and WT-T (* *p* < 0.05, ** *p* < 0.01, *** *p* < 0.001).

Drought stress significantly influenced osmolyte accumulation and chlorophyll content in Arabidopsis, with clear differences among CK, WT-T, *LTS-PYL* overexpression, and *LTS-PYL* genome-edited lines (Figure 6). Proline levels increased significantly in WT-T, *OELTS-PYL-1-T*, and *OELTS-PYL-2-T*, whereas the genome-edited lines showed no significant change compared with CK (Figure 6A). Quantitatively, proline content increased by approximately 58% and 53% in *OELTS-PYL-1-T* and *OELTS-PYL-2-T*, respectively, while it decreased by 20% and 16% in *gelts-pyl-1* and *gelts-pyl-2* relative to WT-T, indicating that *LTS-PYL* activation markedly enhances osmotic adjustment under drought stress.

Sugar content increased significantly in all genotypes compared with CK, with the highest accumulation observed in the *LTS-PYL* overexpression lines (Figure 6B). Specifically, sugar levels were 37% and 46% higher in *OELTS-PYL-1-T* and *OELTS-PYL-2-T*, respectively, relative to WT-T. In contrast, the genome-edited lines exhibited markedly reduced sugar accumulation, with 6% and 11% decreases in *gelts-pyl-1* and *gelts-pyl-2* compared to WT-T. Sucrose content was also substantially elevated in the overexpression lines (Figure 6C). *OELTS-PYL-1-T* and *OELTS-PYL-2-T* accumulated 29% and 32% more sucrose than CK and 111% and 100% more than WT-T. In contrast, *gelts-pyl-1* and *gelts-pyl-2* contained 30% and 15% less sucrose compared with WT-T, suggesting impaired osmotic balance in the absence of functional *LTS-PYL* during drought.

Chlorophyll levels declined in all genotypes under drought stress; however, the reduction was far more pronounced in the genome-edited lines (Figure 6D). Chlorophyll content decreased by 33% and 40% in *gelts-pyl-1* and *gelts-pyl-2* compared with CK. In contrast, *OELTS-PYL-1-T* and *OELTS-PYL-2-T* exhibited only 9% and 20% reductions, indicating their enhanced ability to maintain photosynthetic integrity during drought. These results indicate that *LTS-PYL* overexpression significantly improves osmotic adjustment and chlorophyll integrity under drought stress, while genome editing that disrupts *LTS-PYL* function leads to impaired drought stress.

### 3.7. Correlation Analysis of Physiological, Biochemical, and Gene Expression Parameters

**Figure 7:**
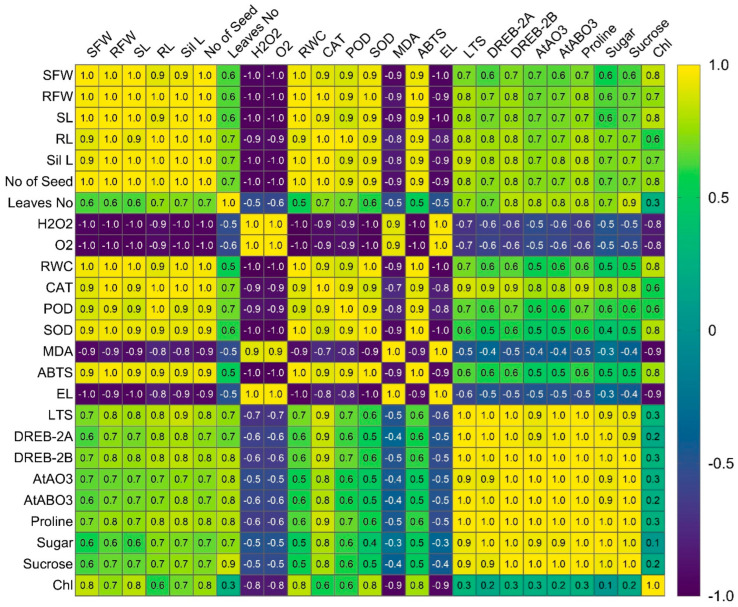
Correlation matrix illustrating the relationship among growth parameters, osmolytes, ROS markers, antioxidant enzyme activities, chlorophyll activity, chlorophyll content, and drought responsive gene expression level in Arabidopsis under drought stress. Positive and negative correlations are represented on a scale from 1.0 to −1.0, respectively, with color intensity indicating the strength of association.

A comprehensive correlation analysis was performed to examine the relationship among growth traits, osmolyte level, oxidative stress markers, antioxidant enzyme activities, and drought responsive gene expression (Figure 7). Growth traits showed strong positive correlation with osmolytes, including proline, sugar, sucrose, and drought-responsive genes, indicating their contribution to maintaining plant vigor during drought. In contrast, ROS markers (H_2_O_2_, O_2_^−^·), were strongly negatively correlated with growth parameters, osmolytes, and chlorophyll levels, reflecting the negative impact of oxidative damage. Antioxidant enzymes (CAT, POD, SOD) displayed strong positive correlations with each other and a negative correlation with ROS markers, highlighting the coordinated role of ROS detoxification. *LTS-PYL* expression showed positive associations with osmolytes, antioxidant activity, and key drought responsive genes, supporting its involvement in enhancing drought tolerance, while negative correlations with ROS and damage indicators further emphasized its protective role.

## 4. Discussion

Drought stress in plants is reduced by complex regulatory network including hormonal signaling, osmotic adjustment, antioxidant defense, and transcriptional reprograming. In this study, we identified *LTS-PYL* as a potent positive regulator of drought adaptation in Arabidopsis, as demonstrated by the contrasting phenotypes of overexpression and CRISPR-Cas9 genome edited lines. The enhanced growth, reproductive, success, physiological resilience observed in *LTS-PYL* overexpression plants together with the severe drought sensitivity of genome edited lines, highlighting the role of this PYR/PYL/RCAR-domain containing gene in coordinating ABA-mediated stress responses. Our finding demonstrated that *LTS-PYL* not only strengthens ABA signaling but also integrates ROS detoxification, osmolyte accumulation, and activation of core drought-responsive transcriptional pathways, collectively conferring superior drought tolerance.

PYR/PYL/RCAR-domain proteins are a class of abscisic acid (ABA) receptors that play central roles in integrating environmental stress signals with plant growth and development [20]. In this study, we demonstrate that *LTS-PYL* not only function as an ABA receptor but also functions as a positive regulator of ABA signaling, coordinating growth maintenance, drought adaptation, and reproductive performance. Functional analyses using *LTS-PYL* overexpression and genome-edited lines revealed that *LTS-PYL* overexpression significantly enhanced endogenous ABA accumulation and upregulated the expression of key ABA and drought-responsive genes, including *DREB2A*, *DREB2B*, *AtAO3*, and *AtABO3*, whereas disruption of *LTS-PYL* compromised these responses (Figure 5). These findings suggest that *LTS-PYL* participates in a positive feedback loop in which ABA perception reinforces ABA biosynthesis, thereby sustaining signaling during prolonged drought. Such feedback regulation is essential for maintaining stress responses over time. Unlike generalized activation of ABA signaling, which often leads to growth inhibition, *LTS-PYL* over expression conferred drought tolerance while preserving vegetative growth and reproductive output [21], indicating a regulatory role that fine-tunes stress responses rather than enforcing growth arrest. Conversely, the impaired germination, reduced seedling biomass, and severe inhibition of root growth observed in *gelts-pyl* mutants (Figure 1), indicate that a basal level of *LTS-PYL* activity is required for optimal ABA signaling even under non-stress conditions [22]. Consistent with the established role of ABA in early developmental regulation, particularly during seedling establishment [23], the contrasting phenotypes between overexpression and genome-edited lines suggest that *LTS-PYL* contributes to maintaining ABA signaling homeostasis, enabling normal growth while preserving sensitivity to environmental cues. This regulatory balance becomes especially critical under drought stress, where *LTS-PYL* overexpression plants maintained sustained shoot and root growth, whereas genome-edited lines exhibited severe growth retardation and premature senescence. ABA signaling operates in a dosage-dependent manner, and excessive ABA perception is known to suppress plant growth [24]. The ability of *LTS-PYL* overexpression lines to sustain biomass accumulation therefore suggests that this receptor optimizes ABA signaling intensity, enabling activation of protective responses without compromising carbon assimilation or developmental processes. In contrast, the pronounced drought sensitivity of *gelts-tyl* mutants reflect a failure to effectively engage ABA-mediated adaptive mechanisms, resulting in impaired growth maintenance and stress resilience. Notably, reproductive development is particularly sensitive to drought stress [25,26], and the preservation of silique length and seed number in *LTS-PYL* overexpression lines (Figure 2), highlights the importance of this receptor in safeguarding reproductive success under drought condition. Our study suggested that maintenance of reproductive traits improved assimilate allocation and hormonal coordination during drought stress, whereas the sever reproductive defects observed in *gelts-pyl* plants highlights the heightened vulnerability of plants lacking functional *LTS-PYL*.

Drought-induced oxidative stress is a major cause of cellular damage and growth inhibition [27]. In this study, *LTS-PYL* overexpression markedly restricted the accumulation of ROS including H_2_O_2_ and O_2_^•−^, while genome-edited lines accumulated excessive ROS under drought stress (Figure 3). The reduced accumulation of ROS in the overexpression lines was accompanied by improved relative water contents, suggesting that *LTS-PYL* coordinated water status and redox balance. A recent study demonstrated that overexpression of *PYL9* in both rice and *Arabidopsis* significantly enhanced drought tolerance through increased ABA accumulation and suppression of excessive ROS production [28]. The authors further reported that elevated ABA levels in *PYL9*-overexpressing plants promoted stomatal closure under drought conditions, leading to improved relative water content and water-use efficiency. This enhanced water status was accompanied by reduced oxidative damage and lipid peroxidation, highlighting the role of *PYL9*-mediated ABA signaling in coordinating water conservation and redox homeostasis during drought stress. The enhanced drought tolerance in *LTS-PYL* overexpression lines was further supported by their strengthened antioxidant defense system. Our results showed that *LTS-PYL* overexpression significantly enhanced the activities of CAT, POD, SOD, and ABTS, while in parallel reducing malondialdehyde (MDA) accumulation and electrolyte leakage, thereby preventing oxidative damage to cellular components (Figure 4). In contrast, the genome-edited lines exhibited the opposite trend, displaying reduced antioxidant capacity and increased membrane damage compared with the overexpression lines. Reduced MDA accumulation and lower electrolyte leakage in overexpression lines demonstrate improved membrane stability [29], which is essential for maintaining cellular integrity, ion homoestasis, and metabolic activity under stress. In contrast, increased lipid peroxidation and membrane damage in genome-edited lines reflect uncontrolled oxidative stress resulting from compromised ABA signaling.

Osmolyte accumulation represents a critical adaptive response to drought stress [30], and in the present study, *LTS-PYL* overexpression markedly enhanced the accumulation of proline, soluble sugars, and sucrose under water-deficit conditions (Figure 6). Drought stress is known to trigger the accumulation of various sugars, resulting in alterations in source–sink relationships in plants [31]. Sugars such as sucrose, glucose, fructose, and raffinose act as key osmolytes and osmoprotectants, contributing to osmotic adjustment, energy supply, and the stabilization of cellular structures. Their accumulation is tightly regulated at the transcriptional level, particularly through sugar transporter genes [32,33]. Both endogenous sugar accumulation and exogenous sugar application have been shown to enhance drought tolerance by alleviating the adverse effects of water deficit on plant growth and development [34,35]. Similarly, proline plays a pivotal role in maintaining cellular integrity by protecting proteins and membranes and by scavenging ROS under stress conditions [36,37]. Under drought stress, proline accumulation is driven by the activation of its biosynthetic pathways and the concomitant suppression of its degradation [30]. Previous studies have also demonstrated that exogenous application of proline mitigates the detrimental effects of drought on plant growth and development [36]. Consistent with these findings, our results demonstrate that *LTS-PYL* overexpression significantly promoted sugar and proline accumulation under drought stress, suggesting improved osmotic adjustment, cellular protection, and ROS detoxification in overexpression lines. In contrast, the reduced accumulation of osmolytes in genome-edited *gelts-pyl* lines indicates impaired metabolic reprogramming resulting from the loss of *LTS-PYL* function. In line with enhanced metabolic and redox homeostasis, overexpression lines maintained higher chlorophyll content under drought stress, reflecting delayed senescence and sustained photosynthetic capacity. Conversely, accelerated chlorophyll degradation observed in *gelts-pyl* mutants suggests early stress-induced senescence driven by oxidative damage and hormonal imbalance.

**Figure 8:**
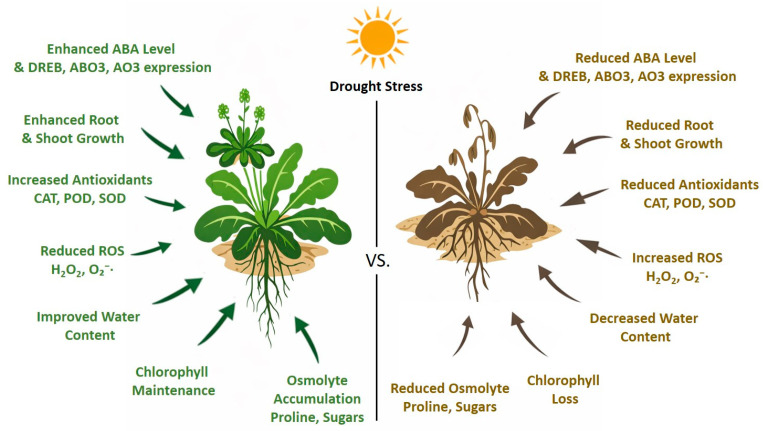
Model illustrating the role of *LTS-PYL* in regulating drought-stress tolerance in Arabidopsis.

This study identifies *LTS-PYL* as a central positive regulator of drought tolerance, functioning at the intersection of ABA perception, redox homeostasis, osmotic adjustment, and transcriptional reprogramming. Using complementary overexpression and CRISPR-Cas9 genome-edited *Arabidopsis thaliana* lines, we demonstrated that *LTS-PYL* fine-tunes ABA signaling strength to activate protective stress responses while preserving vegetative growth and reproductive success. Importantly, *LTS-PYL*-mediated drought adaptation is achieved through coordinated regulation of ROS detoxification, osmolyte accumulation, and ABA-responsive-genes expression, highlighting a regulatory mechanism that balances stress resistance with growth maintenance. A graphical abstract is provided to schematically summarize the LTS-PYL-mediated regulatory network integrating ABA signaling, redox homeostasis, osmotic adjustment, and transcriptional reprogramming during drought stress (Figure 8).

## 5. Conclusions

In conclusion, our findings establish *LTS-PYL* as a key ABA receptor that optimizes drought-responsive signaling networks without imposing growth penalties. *LTS-PYL* enhances drought tolerance by integrating ABA biosynthesis and signaling, antioxidant defense, osmotic adjustment, and transcriptional control, thereby sustaining cellular integrity, photosynthetic capacity, and reproductive performance under water-deficit conditions. Conversely, the severe drought sensitivity of genome-edited lines showed the essential role of *LTS-PYL* in maintaining ABA signaling homeostasis and metabolic resilience. Together, these results provide new mechanistic insight into PYL-mediated drought adaptation and identify *LTS-PYL* as a promising molecular target for engineering drought-resilient crops.

## Data Availability

The original contributions presented in this study are included in the article/Supplementary Material. Further inquiries can be directed to the corresponding authors.

## Supplementary Materials

The following supporting information can be downloaded at: https://www.mdpi.com/article/10.3390/antiox15020178/s1, Supplemental File S1: Locus, name, CDS sequence and functional annotation of LTS-PLY genes; Supplemental File S2: List of genes primers and guide RNAs; Supplemental File S3: PCR8/GW/TOPO used as an entry vector for *LTS-PYL*; Supplemental File S4: pEarleyGate103 used as binary vector; Supplemental File S5: CRISPR/Cas9 expression vector pRGEB32.

## References

[1] Kalleku J.N., Ihsan S., Al-Azzawi T.N.I., Khan M., Hussain A., Chebitok F., Das A.K., Moon Y.S., Mun B.G., Lee I.J. (2024). Halotolerant *Pseudomonas koreensis* S4T10 mitigate salt and drought stress in *Arabidopsis thaliana*. Physiol. Plant..

[2] Muhammad Aslam M., Waseem M., Jakada B.H., Okal E.J., Lei Z., Saqib H.S.A., Yuan W., Xu W., Zhang Q. (2022). Mechanisms of abscisic acid-mediated drought stress responses in plants. Int. J. Mol. Sci..

[3] Akbudak M.A., Filiz E., Kontbay K. (2018). DREB2 (dehydration-responsive element-binding protein 2) type transcription factor in sorghum (*Sorghum bicolor*): Genome-wide identification, characterization and expression profiles under cadmium and salt stresses. 3 Biotech.

[4] Mizoi J., Ohori T., Moriwaki T., Kidokoro S., Todaka D., Maruyama K., Kusakabe K., Osakabe Y., Shinozaki K., Yamaguchi-Shinozaki K. (2013). GmDREB2A; 2, a canonical DEHYDRATION-RESPONSIVE ELEMENT-BINDING PROTEIN2-type transcription factor in soybean, is posttranslationally regulated and mediates dehydration-responsive element-dependent gene expression. Plant Physiol..

[5] Khan M., Imran Q.M., Shahid M., Mun B.-G., Lee S.-U., Khan M.A., Hussain A., Lee I.-J., Yun B.-W. (2019). Nitric oxide-induced AtAO3 differentially regulates plant defense and drought tolerance in *Arabidopsis thaliana*. BMC Plant Biol..

[6] Fujii H., Zhu J.-K. (2009). Arabidopsis mutant deficient in 3 abscisic acid-activated protein kinases reveals critical roles in growth, reproduction, and stress. Proc. Natl. Acad. Sci. USA.

[7] Umezawa T., Sugiyama N., Mizoguchi M., Hayashi S., Myouga F., Yamaguchi-Shinozaki K., Ishihama Y., Hirayama T., Shinozaki K. (2009). Type 2C protein phosphatases directly regulate abscisic acid-activated protein kinases in Arabidopsis. Proc. Natl. Acad. Sci. USA.

[8] Vlad F., Rubio S., Rodrigues A., Sirichandra C., Belin C., Robert N., Leung J., Rodriguez P.L., Lauriere C., Merlot S. (2009). Protein phosphatases 2C regulate the activation of the Snf1-related kinase OST1 by abscisic acid in Arabidopsis. Plant Cell.

[9] Hirayama T., Umezawa T. (2010). The PP2C–SnRK2 complex: The central regulator of an abscisic acid signaling pathway. Plant Signal. Behav..

[10] Yu J., Yang L., Liu X., Tang R., Wang Y., Ge H., Wu M., Zhang J., Zhao F., Luan S. (2016). Overexpression of poplar pyrabactin resistance-like abscisic acid receptors promotes abscisic acid sensitivity and drought resistance in transgenic Arabidopsis. PLoS ONE.

[11] Li Q., Shen C., Zhang Y., Zhou Y., Niu M., Wang H.-L., Lian C., Tian Q., Mao W., Wang X. (2023). PePYL4 enhances drought tolerance by modulating water-use efficiency and ROS scavenging in Populus. Tree Physiol..

[12] Clough S.J., Bent A.F. (1998). Floral dip: A simplified method for Agrobacterium-mediated transformation of *Arabidopsis thaliana*. Plant J..

[13] Park J.-R., Kim E.-G., Jang Y.-H., Jan R., Farooq M., Ubaidillah M., Kim K.-M. (2022). Applications of CRISPR/Cas9 as new strategies for short breeding to drought gene in rice. Front. Plant Sci..

[14] Sadaqat S., Awais M., Rao A.Q. (2025). Functional Characterization of Arabidopsis PQT3 homolog in cotton reveals as a potential candidate for Redox Homeostasis and Abiotic Stress Resistance. Plant Stress.

[15] Khan J., Elsharkawy E., Fu Y., Jan R., Kim K.-M. (2025). Melatonin alleviates lead-induced stress in rice through physiological regulation and molecular defense mechanisms. Sci. Rep..

[16] Jan R., Asaf S., Lubna, Farooq M., Asif S., Khan Z., Park J.-R., Kim E.-G., Jang Y.-H., Kim K.-M. (2024). Augmenting rice defenses: Exogenous calcium elevates GABA levels against WBPH infestation. Antioxidants.

[17] Jan R., Khan M.A., Asaf S., Lubna, Lee I.-J., Kim K.-M. (2021). Over-expression of chorismate mutase enhances the accumulation of salicylic acid, lignin, and antioxidants in response to the white-backed planthopper in rice plants. Antioxidants.

[18] Li R., Jiang M., Song Y., Zhang H. (2021). Melatonin alleviates low-temperature stress via ABI5-mediated signals during seed germination in rice (*Oryza sativa* L.). Front. Plant Sci..

[19] Jan R., Khan M.A., Asaf S., Lubna, Lee I.-J., Kim K.M. (2019). Metal resistant endophytic bacteria reduces cadmium, nickel toxicity, and enhances expression of metal stress related genes with improved growth of Oryza sativa, via regulating its antioxidant machinery and endogenous hormones. Plants.

[20] Chen Y., Feng L., Wei N., Liu Z.-H., Hu S., Li X.-B. (2017). Overexpression of cotton PYL genes in Arabidopsis enhances the transgenic plant tolerance to drought stress. Plant Physiol. Biochem..

[21] Mega R., Abe F., Kim J.-S., Tsuboi Y., Tanaka K., Kobayashi H., Sakata Y., Hanada K., Tsujimoto H., Kikuchi J. (2019). Tuning water-use efficiency and drought tolerance in wheat using abscisic acid receptors. Nat. Plants.

[22] Pizzio G.A., Rodriguez L., Antoni R., Gonzalez-Guzman M., Yunta C., Merilo E., Kollist H., Albert A., Rodriguez P.L. (2013). The PYL4 A194T mutant uncovers a key role of PYR1-LIKE4/PROTEIN PHOSPHATASE 2CA interaction for abscisic acid signaling and plant drought resistance. Plant Physiol..

[23] Finkelstein R.R., Gampala S.S., Rock C.D. (2002). Abscisic acid signaling in seeds and seedlings. Plant Cell.

[24] Rosales M.A., Maurel C., Nacry P. (2019). Abscisic acid coordinates dose-dependent developmental and hydraulic responses of roots to water deficit. Plant Physiol..

[25] Saini H.S. (1997). Effects of water stress on male gametophyte development in plants. Sex. Plant Reprod..

[26] Saini H.S., Westgate M.E. (1999). Reproductive development in grain crops during drought. Adv. Agron..

[27] Mittler R. (2002). Oxidative stress, antioxidants and stress tolerance. Trends Plant Sci..

[28] Zhao Y., Chan Z., Gao J., Xing L., Cao M., Yu C., Hu Y., You J., Shi H., Zhu Y. (2016). ABA receptor PYL9 promotes drought resistance and leaf senescence. Proc. Natl. Acad. Sci. USA.

[29] Blokhina O., Virolainen E., Fagerstedt K.V. (2003). Antioxidants, oxidative damage and oxygen deprivation stress: A review. Ann. Bot..

[30] Haghpanah M., Hashemipetroudi S., Arzani A., Araniti F. (2024). Drought tolerance in plants: Physiological and molecular responses. Plants.

[31] Kaur H., Manna M., Thakur T., Gautam V., Salvi P. (2021). Imperative role of sugar signaling and transport during drought stress responses in plants. Physiol. Plant..

[32] Ghosh U.K., Islam M.N., Siddiqui M.N., Khan M.A.R. (2021). Understanding the roles of osmolytes for acclimatizing plants to changing environment: A review of potential mechanism. Plant Signal. Behav..

[33] Rosa M., Prado C., Podazza G., Interdonato R., González J., Hilal M., Prado F. (2009). Soluble sugars. Plant Signal. Behav..

[34] Wang X., Guo C., Peng J., Li C., Wan F., Zhang S., Zhou Y., Yan Y., Qi L., Sun K. (2019). ABRE-BINDING FACTORS play a role in the feedback regulation of ABA signaling by mediating rapid ABA induction of ABA co-receptor genes. New Phytol..

[35] Ahmad F., Singh A., Kamal A. (2020). Osmoprotective role of sugar in mitigating abiotic stress in plants. Protective Chemical Agents in the Amelioration of Plant Abiotic Stress: Biochemical and Molecular Perspectives.

[36] El Moukhtari A., Cabassa-Hourton C., Farissi M., Savouré A. (2020). How does proline treatment promote salt stress tolerance during crop plant development?. Front. Plant Sci..

[37] Hayat S., Hayat Q., Alyemeni M.N., Wani A.S., Pichtel J., Ahmad A. (2012). Role of proline under changing environments: A review. Plant Signal. Behav..

