# Supplementary material for: Functional Analysis of LTS-PYL in Modulating Plant Drought Responses
Source: Antioxidants (Basel). 2026 Jan 30;15(2):178. doi: 10.3390/antiox15020178 (PMC12938716; doi:10.3390/antiox15020178)

Supplemental File S1. Locus, name, CDS sequence and functional annotation of LTS-PLY genes

Locus: AT4G32870 474BP (Lipid Transport Superfamily LTS ply5)

CDS sequence
ATGGGAACGGAAACGGTGTCGTTTAAGTGGGAGGGTAAGAAAGTCGCTCAAGTC
AACGGTGTCACGGCTGAGAAAGTTTGGTCGGTTTTTTCTGACTTCTGCAACGTCA
AGAGTGGTTCCCATCAGTCGATACGTGTTACCGAGTCCAAGGAACCGATGGTGT
ACCGGGTCTGATCCGATACTGCTCCACCACCAAAACCAAGGAAGAGGGAAGCAG
GTGGGCTAAAGAGAAGCTGGTCAAGATCGATCCCATTGGACGGTGTTTGAGCTA
TGAGATCCTCGAGAATAACGTAGGGTTTAGATCTTACGTGGCGACAGTCCAAGT
AACGCCAGTGGACGGTGAAGATCAAGTGTCTCGAATCGAGTGGTCGTTTGTTGCC
GATCCTGTTGATGGTTGGAAGAAGGAAGACCTCGAATCATACGTTGACTTTTGCC
TTCAACATATGGCCAACAAAATGGAACTCAATCTGTAA

Description: Polyketide cyclase/dehydrase and lipid transport superfamily protein; (source:Araport11)

GO Biological Process	involved in	abscisic acid-activated signaling pathway, regulation of protein serine/threonine phosphatase activity
GO Cellular Component	located in	cytoplasm, nucleus
GO Molecular Function	has	abscisic acid binding, protein phosphatase inhibitor activity, signaling receptor activity
Growth and Developmental Stages	expressed during	LP.02 two leaves visible stage, LP.04 four leaves visible stage, LP.06 six leaves visible stage, LP.08 eight leaves visible stage, LP.10 ten leaves visible stage, LP.12 twelve leaves visible stage, flowering stage, petal differentiation and expansion stage, vascular leaf senescent stage
Plant structure	expressed in	carpel, cauline leaf, collective leaf structure, cotyledon, flower, guard cell, hypocotyl, inflorescence meristem, leaf apex, leaf lamina base, petal, petiole, pollen, root, sepal, shoot apex, shoot system, stamen, stem, vascular leaf

Supplemental File S2. List of genes primers and guide RNAs.

S.No	Name	Forward primer	Reverse primer
1	LTS-PYL	ATGGGAACGGAAACGGTGTC	TTACAGATTGAGTTCCATTTTGTTGG
2	DERB-2A	GACCTAAATGGCGACGATGT	TCGAGCTGAAACGGAGGTAT
3	DREB-2B	GATTTCTGTAAAAAGTTTGTTGT	AGCCAATGAACCGTACATAGCGGTA
4	AtAO3	GCTGTGGCGAAGTGTAAATGG	TAACCTGTACCCGTGTTGCC
5	AtABO3	CAGGGATGCGCTAACATCCT	CGGGGATTGGGGGAATCATC
6	sgRNA1	AGTCCAAGGAACCGATGGTG	CACCATCGGTTCCTTGGACT
7	sgRNA2	ATCAAGTGTCTCGAATCGAG	CTCGATTCGAGACACTTGAT

Supplemental File S3. PCR8/GW/TOPO used as an entry vector for *LTS-PYL*

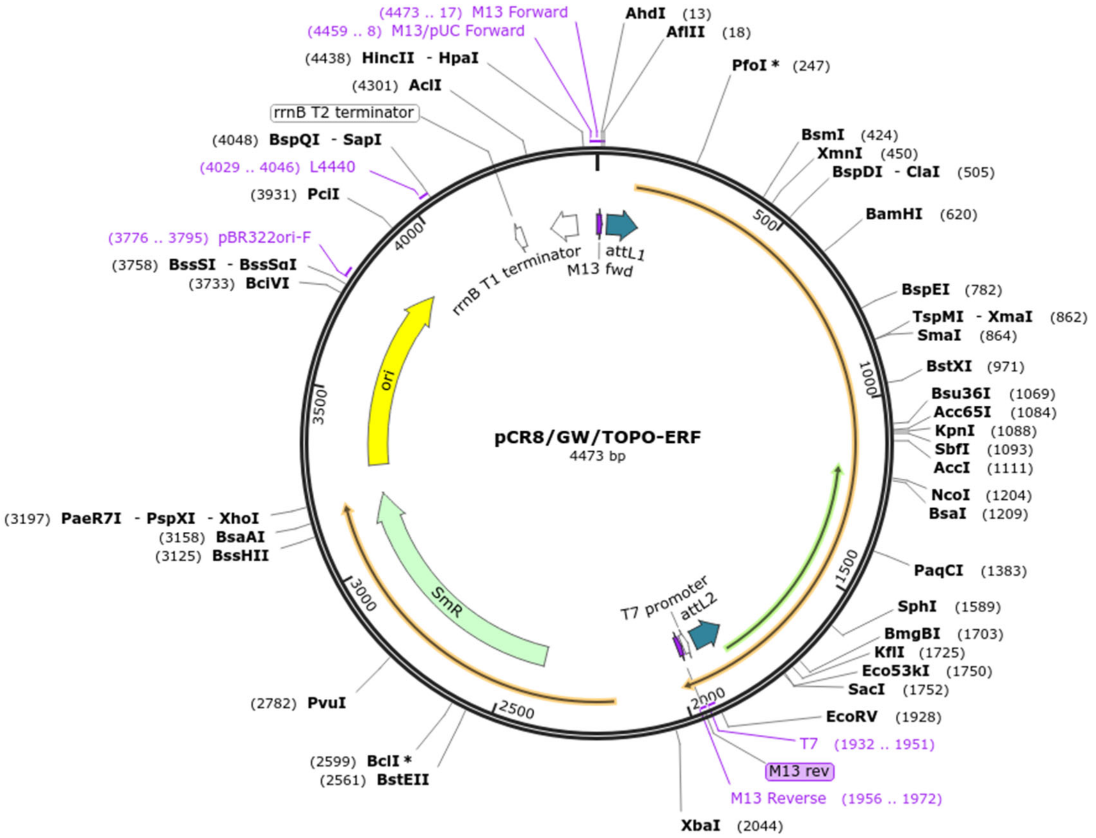

Supplemental File S4. pEarleyGate103 used as binary vector

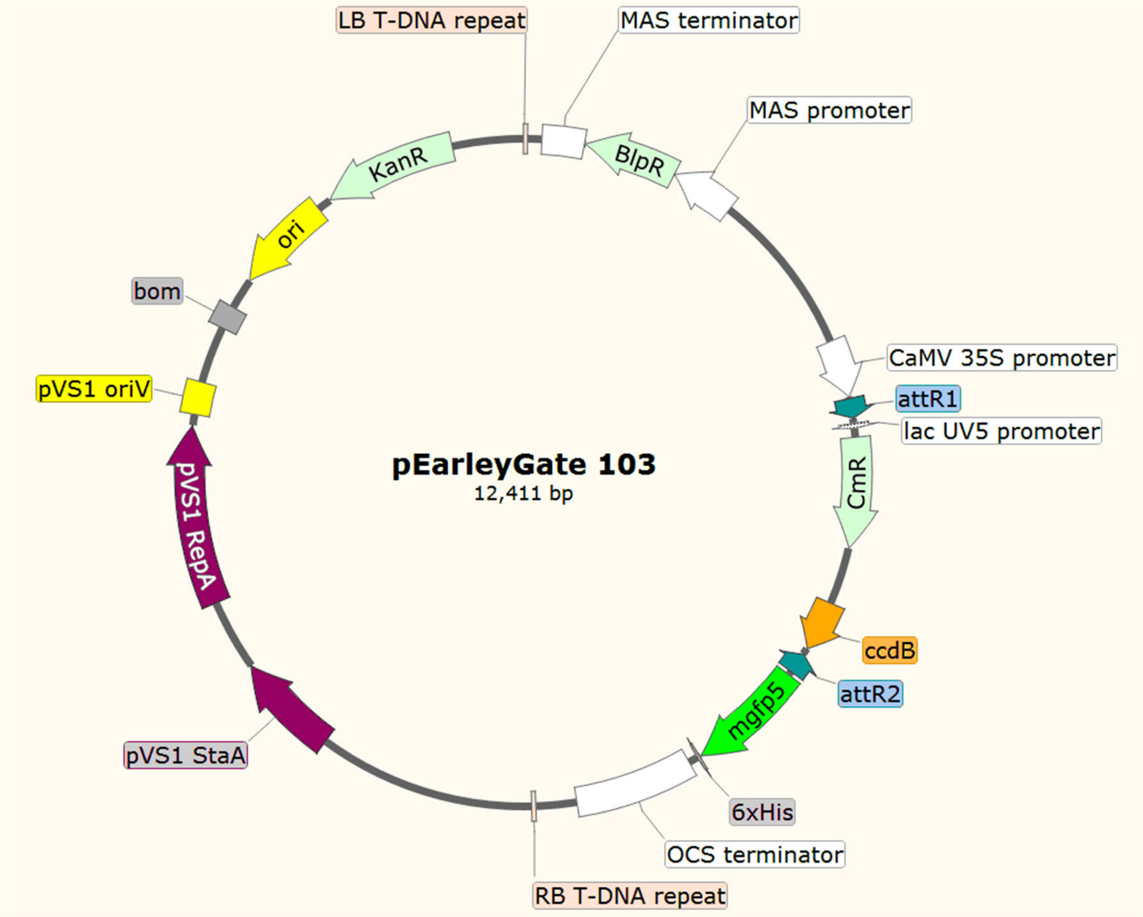

Supplemental File S5. CRISPR/Cas9 expression vector pRGEB32.

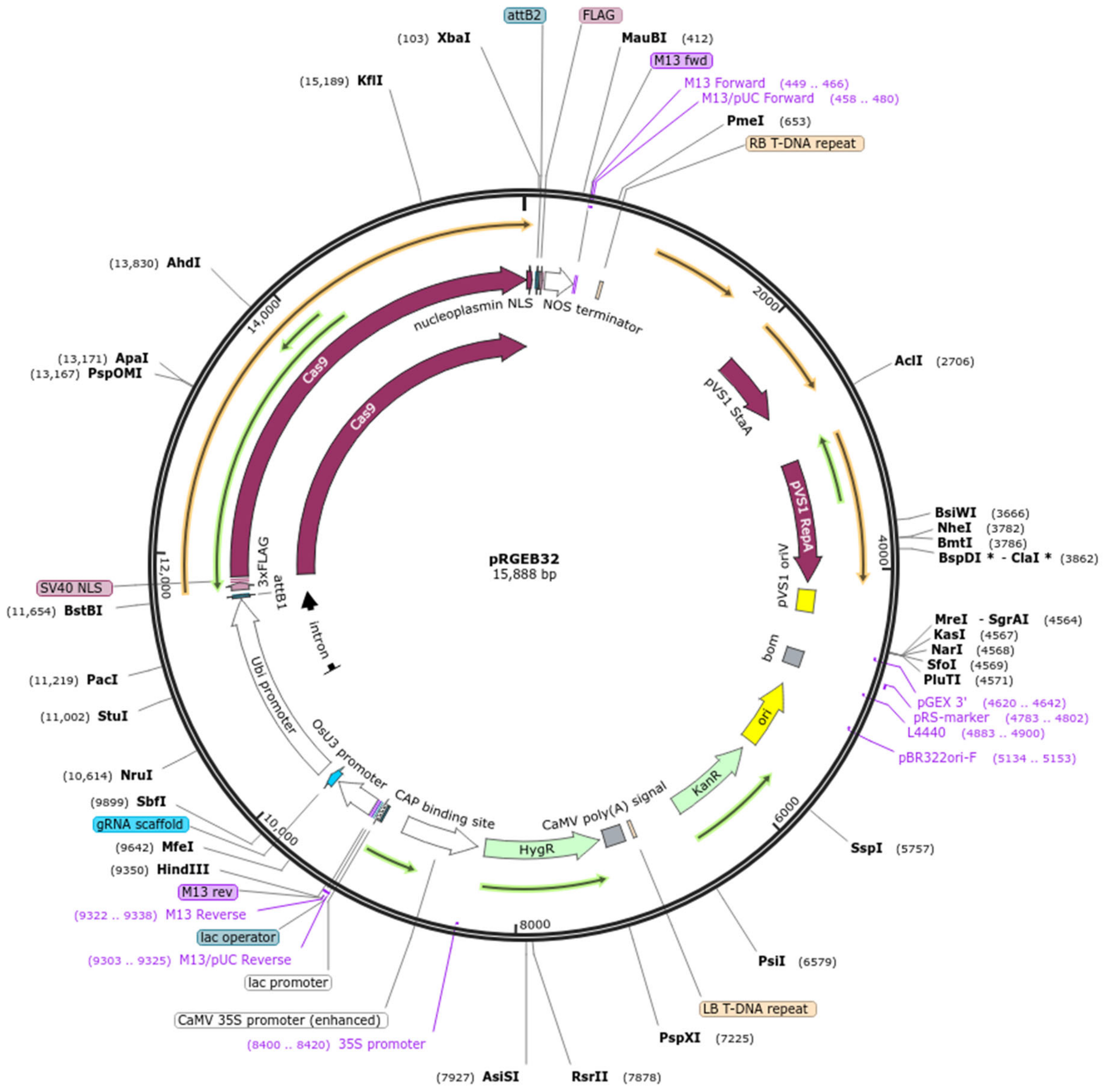

Supplement: Supplementary file 1 [file antioxidants-15-00178-s001.zip › antioxidants-4079944-supplementary.pdf]
